# Imaging Brain Glx Dynamics in Response to Pressure Pain Stimulation: A ^1^H-fMRS Study

**DOI:** 10.3389/fpsyt.2021.681419

**Published:** 2021-07-28

**Authors:** Luke A. Jelen, David J. Lythgoe, Jade B. Jackson, Matthew A. Howard, James M. Stone, Alice Egerton

**Affiliations:** ^1^Institute of Psychiatry, Psychology, and Neuroscience, King's College London, London, United Kingdom; ^2^South London and Maudsley National Health Service (NHS) Foundation Trust, London, United Kingdom; ^3^Medical Research Council (MRC) Cognition and Brain Sciences Unit, University of Cambridge, Cambridge, United Kingdom; ^4^Department of Neuroscience and Imaging, University of Sussex, Brighton, United Kingdom

**Keywords:** functional magnetic resonance spectroscopy, ^1^H-fMRS, glutamate, Glx, glutamine + glutamate, pain

## Abstract

Glutamate signalling is increasingly implicated across a range of psychiatric, neurological and pain disorders. Reliable methodologies are needed to probe the glutamate system and understand glutamate dynamics *in vivo*. Functional magnetic resonance spectroscopy (^1^H-fMRS) is a technique that allows measurement of glutamatergic metabolites over time in response to task conditions including painful stimuli. In this study, 18 healthy volunteers underwent ^1^H-fMRS during a pressure-pain paradigm (8 blocks of REST and 8 blocks of PAIN) across two separate sessions. During each session, estimates of glutamate + glutamine (Glx), scaled to total creatine (tCr = creatine + phosphocreatine) were determined for averaged REST and PAIN conditions within two separate regions of interest: the anterior cingulate cortex (ACC) and dorsal ACC (dACC). A two-way repeated measures analysis of variance determined a significant main effect of CONDITION (*p* = 0.025), with higher Glx/tCr during PAIN compared to REST across combined sessions, in the dACC ROI only. However, increases in dACC Glx/tCr during PAIN compared to REST showed limited reliability and reproducibility across sessions. Future test-retest ^1^H-fMRS studies should examine modified or alternative paradigms to determine more reliable methodologies to challenge the glutamate system that may then be applied in patient groups and experimental medicine studies.

## Introduction

Glutamate, a major excitatory neurotransmitter, is fundamentally involved in normal and abnormal brain function (1). Glutamate plays a central role in a number of pathologies including psychiatric (2), neurological (3), neurodevelopmental (4) and pain disorders (5). Proton magnetic resonance spectroscopy (^1^H-MRS) is a non-invasive in vivo technique that allows the measurement of regional concentrations of brain metabolites, including glutamate, and has proved integral in furthering our understanding of the role of glutamate in health and disease (6). However, a significant limitation of standard ^1^H-MRS is that measures are determined over a single resting (static) time period, preventing dynamic glutamate responses from being studied.

Functional ^1^H-MRS (^1^H-fMRS) is a technique where sequential ^1^H-MRS scans are acquired to measure dynamic changes in metabolite concentrations. There has been particular interest in using ^1^H-fMRS methods to investigate changes in glutamate induced by tasks and other stimuli (7). Compared with standard static ^1^H-MRS glutamate measurements, the signal change detected with ^1^H-fMRS may relate more closely to glutamate involved in neurotransmission (7, 8). ^1^H-fMRS studies have demonstrated significant dynamic increases in glutamate measures in the occipital cortex during visual stimuli presentation (9–13) and in the motor cortex during motor stimuli (14, 15). ^1^H-fMRS studies have also demonstrated significant increases in glutamate measures in healthy controls in the anterior cingulate cortex (ACC) during cognitive tasks including the Stroop task (16–18) and increases in Glx/tCr (glutamate + glutamine referenced to total creatine) in the N-back task (19). However, increases in ACC glutamate measures were not seen in patients with major depressive disorder or schizophrenia during the Stroop task (18), neither were increases in Glx/tCr detected in patients with schizophrenia or bipolar disorder II during the N-back task (19). These previous data emphasise the potential of ^1^H-fMRS to investigate abnormalities in glutamate dynamics associated with psychiatric disorders.

Potential challenges associated with the use of cognitive paradigms include the influence of task-learning effects, and individual or group differences (healthy vs. patient) in motivation or ability to complete the task. Additionally, the magnitude of the glutamate response detected in ^1^H-fMRS cognitive studies is relatively small (8). In contrast, the use of painful stimuli may avoid some of these potential confounds and appear to elicit the largest ^1^H-fMRS glutamate responses (7). ^1^H-fMRS pain studies have demonstrated increases in glutamate and Glx in the ACC during a cold-pressor task (20); an increase in Glx/tCr in the dorsal ACC (dACC) (21), increased concentrations of glutamate and Glx in the dACC (22), an increase in glutamate in the insula (23) following painful heat stimuli; and an increase in Glx/tCr in the insula during dental pain (21). There is some inconsistency in ^1^H-fMRS pain findings with one study reporting reductions in glutamate levels in the dACC following heat pain exposure (24) while another found no change in Glx/tCr in the insula during dental pain (25). Furthermore, none of these ^1^H-fMRS pain studies have employed a test-retest design and the reliability of any elicited ^1^H-fMRS responses has not been described.

Findings from a recent functional magnetic resonance imaging (fMRI) study, that demonstrated robust, reliable estimates of blood oxygen-level-dependent (BOLD) signal and self-reported pain in response to noxious pressure across two separate sessions (26), suggests that pressure as a stimulation modality is an alternative viable method in the study of pain. Similar to thermal heat paradigms (21, 23), experimental pressure pain paradigms allow the ability to control stimulus intensity and delivery accounting for individual pain thresholds and can be practically delivered within an MR environment (26). In this study, we aimed to determine if pressure pain stimulation could be applied to generate reliable changes in a glutamate measure (Glx/tCr) as measured by ^1^H-fMRS. ^1^H-fMRS was employed to examine pressure pain-induced dynamic Glx/tCr responses across two separate sessions in healthy volunteers in the ACC, a region known to be activated during pain perception (27, 28) and the dACC, a region in which previous ^1^H-fMRS studies have reported increases in glutamate, Glx and Glx/tCr during painful stimuli (21, 22). We hypothesised that Glx/tCr would be elevated in these regions during a pressure pain condition relative to a resting passive visual fixation condition. We further hypothesised that changes in Glx/tCr between the rest and pain conditions would be related to subjective pain rating scores.

## Materials and Methods

### Participants

Eighteen healthy subjects (mean age 25 ± 4.74, male/female: 9/9, right/left-handed: 17/1) were recruited via local and online advertisements. Volunteers with a history of psychiatric, neurological, chronic pain condition, or history of hand/thumb trauma were excluded. Volunteers with major medical illness requiring medication, magnetic resonance imaging (MRI) contraindications or history of substance or alcohol abuse were also excluded from the study. Previous work has indicated variability in the pain responses of females depending on the phase of the menstrual cycle (29). Female volunteers completed test- retest sessions within the equivalent phase of consecutive months and those with irregular menstrual cycles were excluded. To minimise the influence of diurnal variations on pain (30, 31), participants were all tested at the same time in the day. Before each visit, participants were required to abstain from paracetamol or non-steroidal anti-inflammatory drugs for 12 h. Prior to any experiments all volunteers were informed about the study procedures, potential risks and provided written informed consent. The study was approved by the local Ethics Committee (Reference HR-18/19-8825).

### Study Sessions

All participants attended four separate sessions. In the first session participants underwent a thresholding procedure for pressure pain stimulation, which took place in a mock scanner environment. This was followed by the first imaging session that took place within 1 week of the thresholding session, where the pressure pain paradigm was administered in an MRI scanner. The third and fourth sessions were repeats of the first and second sessions respectively (i.e., second thresholding and second imaging session), and occurred after an interval of 4 weeks. The second thresholding session was performed to account for potential changes in individuals pain threshold over time. The mean interval between the two imaging sessions was 28.44 days, SD = 3.50. The mean interval between the thresholding and imaging sessions was 3.92 days, SD = 2.56.

### Pressure Pain Thresholding Sessions

Pressure stimuli were applied to the thumbnail of the non-dominant hand using a custom-made, automated, pneumatic, computer-controlled stimulator with a plastic piston that applies pressure via a 1.13 cm^2^ hard rubber probe (26, 32). The thumb was inserted into a cylindrical opening and positioned such that the probe applied pressure directly to the nail bed. To maintain consistency, the same pressure device was used for both the thresholding sessions and the pressure pain paradigm in the imaging sessions.

The pressure pain thresholding procedure (26) involved one ascending series and one randomised series of pressure stimuli. During the ascending series of pressure stimuli, participants first received stimulation at 55 kilopascals (kPa) for 2 s duration. Subsequent pressure stimulations increased in steps of 4 kPa, delivered at 4 s intervals, again each lasting 2 s duration. First, participants were required to inform the investigator when they had reached their minimum pain threshold (pain score > 0 on a pain scale where 0 = “no pain” and 100 = “worst pain imaginable”). Second, participants were asked to inform the investigator when they reached their moderate pain threshold [pain score = 70 (70%)]. These values from the ascending series were then used to compute the magnitude of five different pressure intensities within the range of each subject's minimum and moderate pain thresholds. For example, if a subject's minimum pain threshold was represented by a pressure of 200 kPA and moderate pain threshold [pain score = 70 (70%)] was reached at a pressure of 600 kPA then the randomised series would consist of pressures of 200, 300, 400, 500, and 600 kPa. Subsequently, during the randomised series, each of these five pressure stimulations were delivered three times (15 stimuli in total) for a duration of 2 s in a pseudo-randomised order at 24 s intervals. During each interval participants were required to rate the intensity of their pain on a computerised pain visual analogue scale (VAS) that was presented on screen for 7 s, using their contralateral hand. Finally, a linear function was used to determine each individual's representation of a pain score of 60 (60%), built on the 15 ratings from the randomised series.

### Imaging Sessions

**Figure 1:**
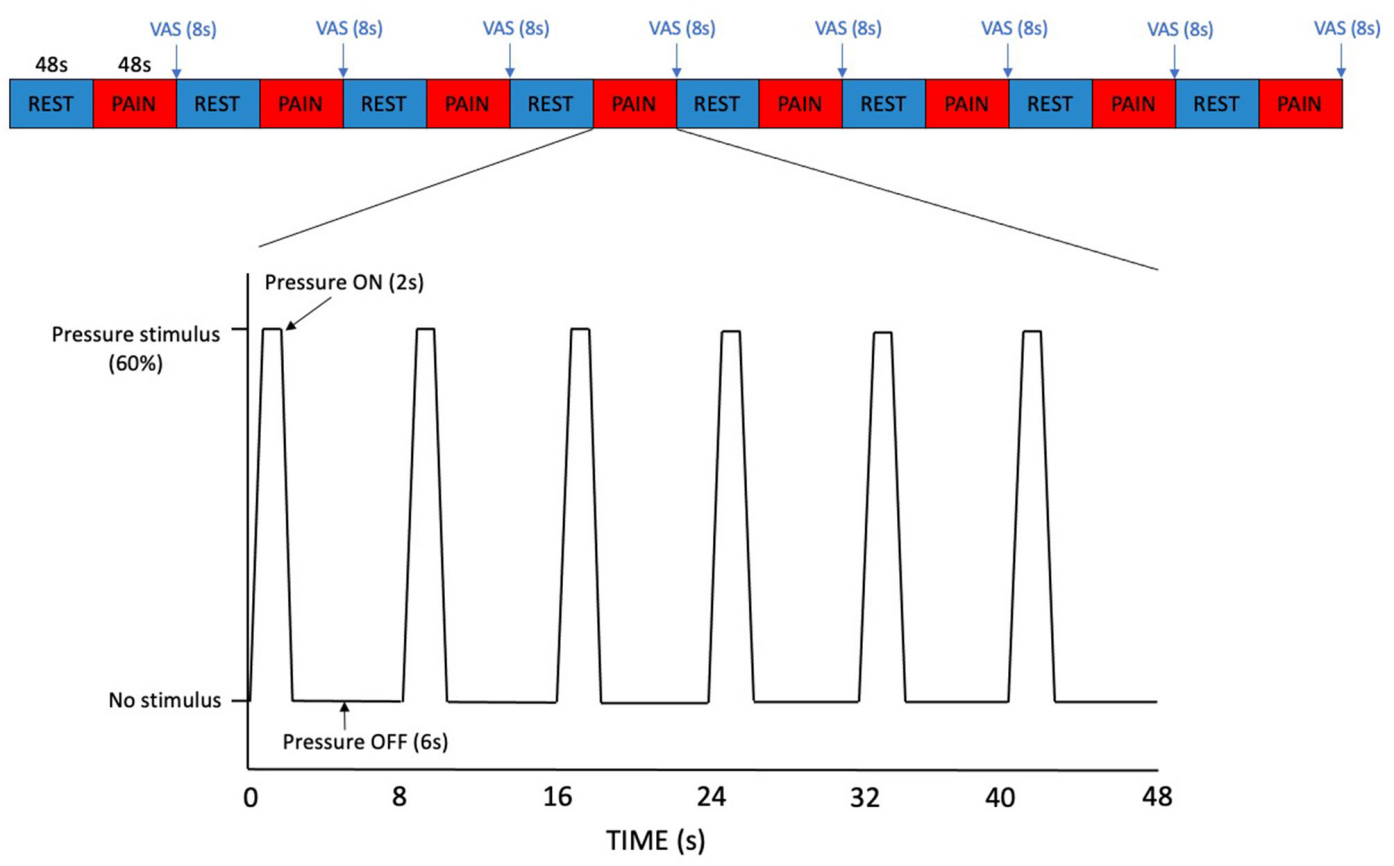
Pressure pain paradigm. Each block of REST and PAIN was 48 s in duration. In the PAIN blocks, each pressure stimulus had a duration of 2 s, separated by a 6 s interval. A VAS pain scale was displayed for 8 s at the end of each PAIN block.

Participants first underwent localiser and structural scans followed by the pressure pain paradigm (Figure 1), utilising the same pressure probe device as used in the thresholding sessions. The paradigm was a block design consisting of sixteen alternating blocks of rest (no stimulation, passive visual fixation) and pressure pain stimuli delivered at each individual's pain score of 60% determined from the previous thresholding session. The rationale for delivering pressure set for a pain score of 60%, rather than 70% (moderate pain threshold), was to account for the repeated pressure stimuli applied in the paradigm and to make the experiment more tolerable for participants. Each block was 48 s in duration. In the pressure pain stimulus block, each pressure stimulus had a duration of 2 s, separated by a 6 s interval and for each stimulus block there were six pressure stimuli in total. At the end of each pressure pain stimuli block, participants were presented with a computerised VAS of the pain scale, as in the thresholding session, but displayed for 8 s. Apart from when the VAS was on screen, a fixation cross was displayed for the rest of the paradigm. The total duration of one run of the pressure pain paradigm was 832 s. Participants completed one run per voxel (both on the non-dominant hand) with a 5-min break to rest their hand, during each of the imaging sessions. The procedure for the second imaging session was identical to the first apart from the pressure pain stimuli delivered that depended on the values determined from each individual's second thresholding session.

### ^1^H-fMRS Data Acquisition

**Figure 2:**
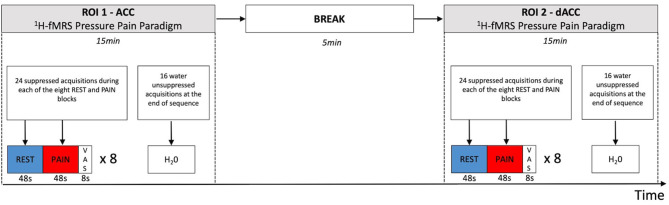
Temporal sequence of ^1^H-fMRS measurements and regions. ^1^H-fMRS measurements were first acquired from ROI 1 (ACC) during the pressure pain paradigm. There was then a 5 min break and subsequently ^1^H-fMRS measurements were acquired from ROI 2 (dACC) during a second run of the same pressure pain paradigm. The ^1^H-fMRS sequence was identical for each ROI (T_R_= 2,000 ms, T_E_ = 105 ms, phase cycle length = 2). Twenty four suppressed acquisitions were acquired during each of the eight REST and PAIN blocks with 16 water unsuppressed acquisitions acquired at the end of the ^1^H-fMRS sequence for each run.

**Figure 3:**
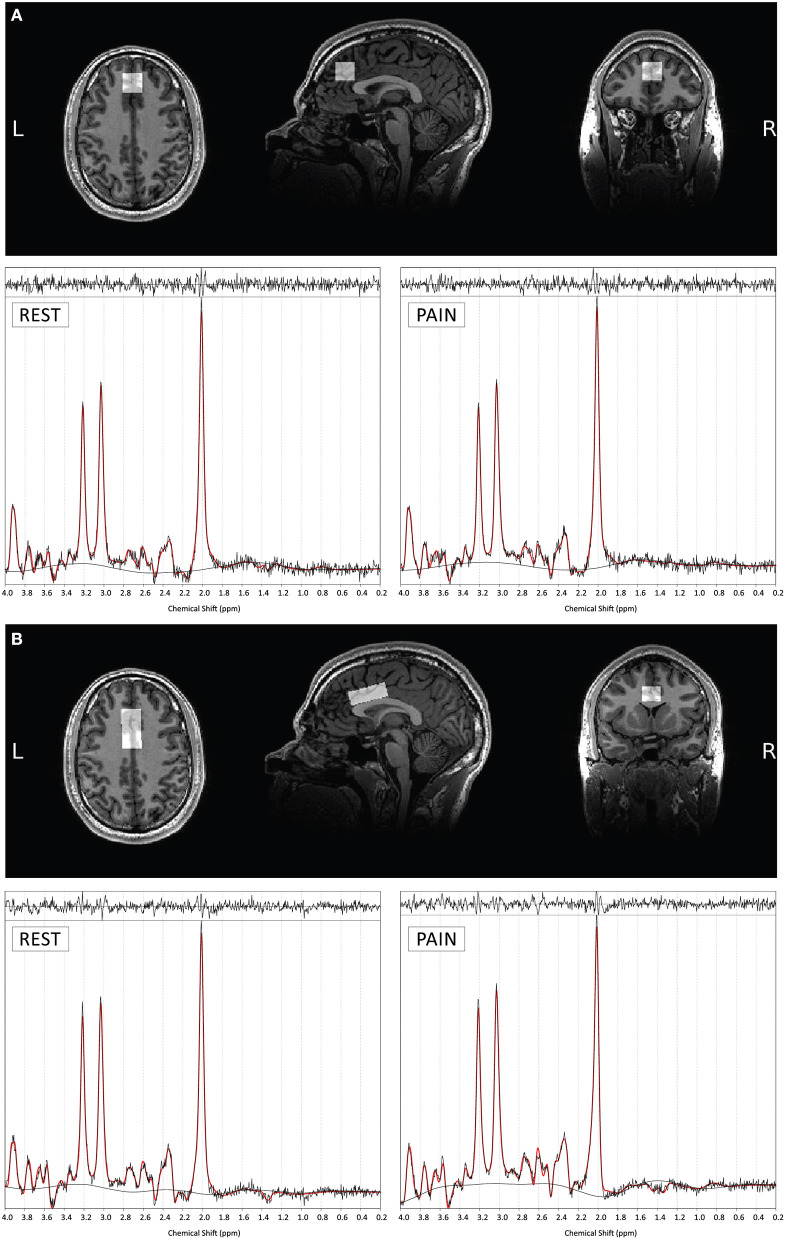
Example voxel positioning and associated ^1^H-fMRS spectra (for a single subject) for ROI 1- ACC **(A)** and ROI 2- dACC **(B)**. The screen shots of the orthogonal views are taken through the center of each plane of each of the voxels. Example TARQUIN output is shown for REST and PAIN conditions with output of the fit (red) overlaid on the acquired spectrum (black). The estimated baseline is displayed under each spectrum in black.

The data were acquired using a GE Discovery MR750 3.0T scanner equipped with a 32-channel receive-only head coil at the Centre for Neuroimaging Sciences, King's College London, UK. A high-resolution structural 3D IR-SPGR sequence (T_R_ = 7.31 ms, T_E_ = 3.02 ms, T_I_ =400 ms, FOV = 270 mm, flip-angle (a) = 11°, matrix size = 256 × 256, slice thickness = 1.2 mm, 196 slices) in the sagittal plane was first acquired for localisation of the spectroscopy voxels. ^1^H-fMRS spectra were acquired in two separate brain regions of interest (ROI) during the pressure pain paradigm (Figure 2). ROI-1 was an anterior cingulate cortex (ACC) region where the voxel measured 20 × 20 × 20 mm with the centre of the voxel placed 16 mm above the genu of the corpus callosum perpendicular to the anterior commissure- posterior commissure line (Figure 3A). This non-rotated ACC voxel aligns with that used in our previous ^1^H-MRS studies in patient groups (33, 34). ROI-2 was a dorsal ACC (dACC) region where the voxel measured 40 × 20 × 15 mm and was positioned at an angle to sit immediately above the corpus callosum, with the anterior edge of the voxel aligned with the anterior edge of the genu of the corpus callosum (Figure 3B). The order of ^1^H-fMRS acquisition was fixed with data collected from ROI-1 (ACC) first and ROI-2 (dACC) second for all participants.

The ^1^H-fMRS spectra were acquired individually throughout the pressure pain paradigm using Point RESolved Spectroscopy (PRESS), with CHEmical Selective Suppression for water suppression and outer volume suppression (OVS) with Very Selective Suppression (VSS) pulses (T_R_= 2,000 ms, T_E_ = 105 ms, phase cycle length= 2, 420 acquisitions, 16 water unsuppressed acquisitions). Before each ^1^H-fMRS scan, shimming was optimised with an auto-prescan performed twice. A modified version of GE's PROBE (proton brain examination) sequence was used to commence the pressure pain paradigm and send a trigger pulse at the start of every T_R_.

### ^1^H-fMRS Analysis

Analysis of ^1^H-fMRS data was performed using the TARQUIN software package, version 4.3.10 (35). The TARQUIN algorithm performed a fully automated fit to the ^1^H-fMRS data using a predefined basis set comprising of the following components: alanine; aspartate; creatine (Cr); gamma-aminobutyric acid; glucose; glutamine; glutathione; glutamate (Glu); glycerophosphorylcholine; myo-inositol; lactate; lipid peaks at 0.9, 1.3a, 1.3b, and 2.0 ppm; macromolecules at 0.9, 1.2, 1.4, and 2.0 ppm; N-acetyl-aspartate (NAA); N-acetyl- aspartateglutamate; phosphorylcholine; phosphocreatine (PCr); scyllo-inositol; and taurine.

^1^H-fMRS spectra acquired during the “REST” and “PAIN” conditions (8 blocks of each) from each ROI were averaged within subjects and estimates of Glx (Glu + glutamine) were determined. Only Glx values were reported as with the T_E_ used in this study glutamate and glutamine may not be reliably differentiated. Averaged ^1^H-fMRS spectra had to meet the following quality control measures to be included in subsequent analysis: (1) Cramer-Rao minimum variance bounds (CRMVB) estimate of the standard deviation of Glx < 20%; (2) signal-to-noise ratio (SNR) > 5; (3) full-width half- maximum (FWHM) < 0.1 ppm and (4) fit quality (Q) < 2.5. No spectra had to be discarded following the quality control process. Total creatine (tCr = Cr + PCr) was used to provide a reference for Glx values in each sub spectrum to alleviate possible confounds of scanner drift that would not be adequately corrected if a single water reference that was acquired at the end of the scan was used instead.

To assess consistency of voxel tissue composition, IR-SPGR images were segmented using SPM12 (http://www.fil.ion.ucl.ac.uk/spm/software/spm12/) into grey matter (GM), white matter (WM) and cerebrospinal fluid (CSF) and mean tissue proportions and standard deviations were determined across subjects and scanning sessions.

### Statistics

Normality of data was examined using Shapiro–Wilk tests. To evaluate *a priori* hypotheses, two-way repeated measures analyses of variance (ANOVAs) were used to examine differences in Glx/tCr, with CONDITION (REST and PAIN) and SESSION (Session A and Session B) as within-subject factors, including CONDITION^*^SESSION interaction terms, for each ROI individually. As future research applications may wish to evaluate Glx/tCr responses during a single scanning session (for example in case-control designs), and to facilitate qualitative comparison with previous research using similar sample sizes that applied only a single session (21, 22), we additionally investigated whether effects of CONDITION reached significance in session A alone, using paired samples *t*-test.

To assess the test-retest reliability across scanning sessions for the change in Glx/tCr in the PAIN compared to REST condition, the intra-class correlation coefficient (ICC), was computed using a two-way mixed effects model, single measurement for absolute agreement alongside the within-subject percentage coefficient of variation (%CV). Test-retest variability (VAR) was also computed to assess reproducibility. Reliability and reproducibility tests was only evaluated where significant main effects of CONDITION were detected.

Exploratory analyses examined for potential adaptation effects for Glx/tCr across individual blocks of the pressure pain paradigm using three-way repeated measures ANOVAs with BLOCK, CONDITION and SESSION as within-subject categorical factors (including BLOCK^*^CONDITION, BLOCK^*^SESSION, CONDITION^*^SESSION and BLOCK^*^CONDITION^*^SESSION interaction terms). These analyses were performed separately for each ROI and all Glx values for individual blocks, including those with CRMVB >20%, were included.

Finally, Pearson's product moment correlation coefficients were used to examine any relationships between ΔGlx/tCr between REST and PAIN conditions and VAS pain scores (averaged within-subject and compared across subjects) for each scanning session. For all analyses, a significance threshold of p < 0.05 was specified. Analyses were carried out using R version 3.6.3 and IBM SPSS Statistics version 27.

## Results

### Pain VAS Scores

The mean stimulus pressures delivered (determined from thresholding sessions) and mean pain VAS scores from the ^1^H-fMRS pressure pain paradigm for each ROI are shown in Table 1. The mean stimulus pressure delivered and mean pain VAS scores for each ROI remained stable with no statistically significant differences across sessions (Table 1). Mean pain VAS scores were higher for the second run, dACC (ROI-2), than the first run, ACC (ROI-1) for both sessions. Mean pain VAS scores across individual PAIN blocks and across sessions for each ROI are shown in Supplementary Figure 1.

**Table 1:** Mean stimulus pressure values and pain VAS scores.

	**SESSION A, mean (SD)**	**SESSION B, mean (SD)**	***p***
Stimulus pressure delivered (kPa) (Determined from thresholding session)	676 (115)	665 (95)	0.58
Average Pain VAS ROI-1 (ACC) (All blocks)	53.3 (10.3)	53.8 (8.4)	0.85
Average Pain VAS ROI-2 (dACC) (All blocks)	59.9 (13.6)	61.3 (13.4)	0.76

*p, p-value pairwise comparisons (alpha = 0.05, two-tailed)*.

### Metabolite Ratio Differences (Pressure Pain Paradigm)

**Figure 4:**
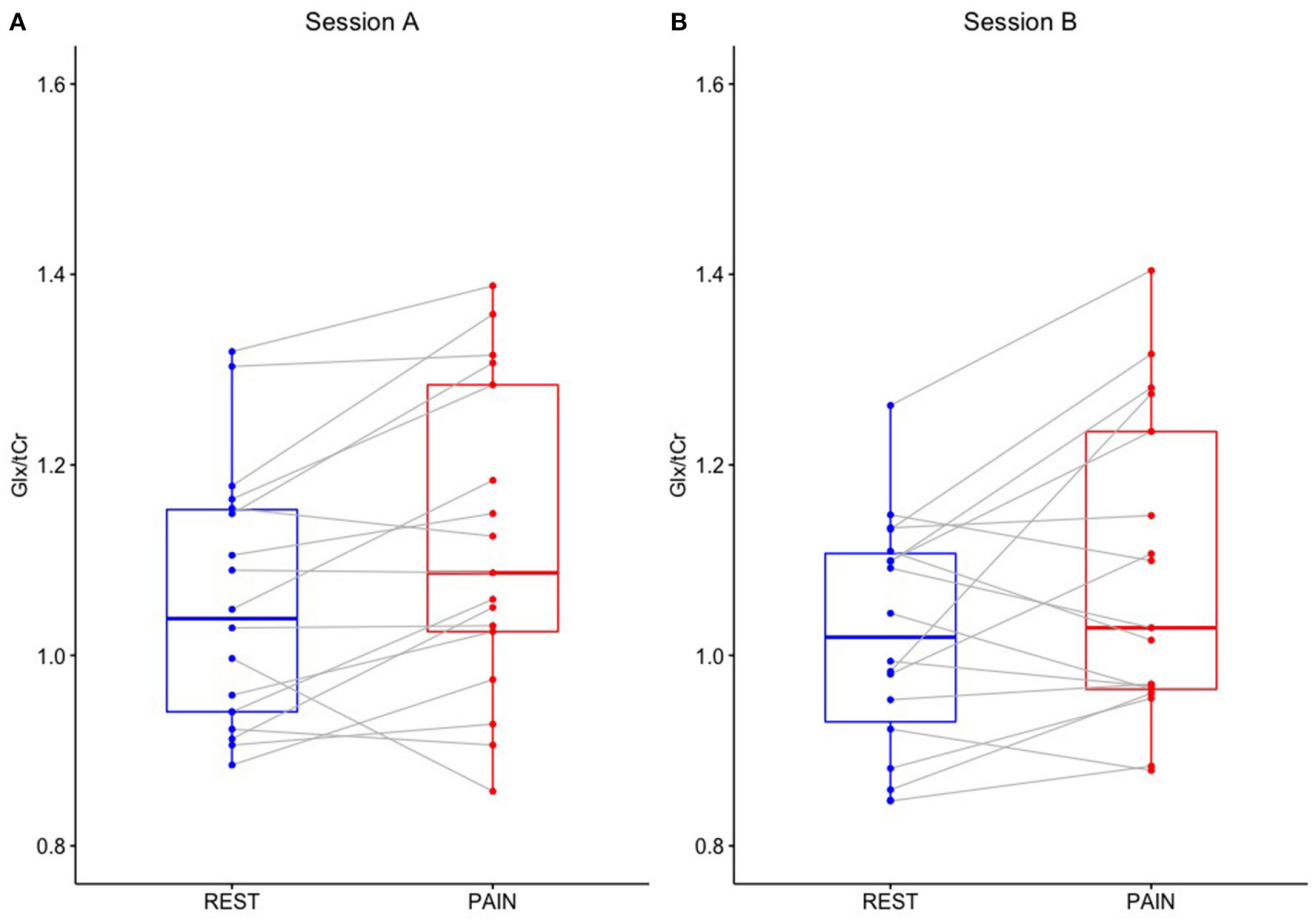
Glx/tCr in the dACC (ROI-2) during REST and PAIN conditions for scanning sessions **(A,B)**.

Two-way repeated measures ANOVA demonstrated a significant main effect of CONDITION for Glx/tCr in the dACC (ROI-2) [*F*_(1,17)_ = 6.075, *p* = 0.025], with higher Glx/tCr during PAIN compared to REST. There was no significant main effect of SESSION [*F*_(1,17)_ = 0.672, *p* = 0.424] and no significant CONDITION ^*^ SESSION interaction [*F*_(1,17)_ = 0.028, *p* = 0.870] for dACC Glx/tCr (Table 2). dACC Glx/tCr during REST and PAIN is presented in Table 3 and Figure 4. The increase in dACC Glx/tCr during PAIN compared to REST did not reach statistical significance for session A alone [*t*_(17)_ = 2.012, *p* = 0.060].

**Table 2:** Two-way ANOVA summary table for Glx/tCr and tCr for each ROI.

**ROI and metabolite**	**Source**	**df**	**MS**	***F***	***p***
ACC (ROI-1) [Glx/tCr]	CONDITION	1	0.015	2.620	0.124
	*Error (CONDITION)*	17	0.006		
	SESSION	1	0.035	2.703	0.119
	*Error (Session)*	17	0.013		
	CONDITION*SESSION	1	0.002	0.202	0.659
	Error (CONDITION * SESSION)	17	0.008		
ACC (ROI-1) [tCr]	CONDITION	1	0.088	0.403	0.534
	*Error (CONDITION)*	17	0.219		
	SESSION	1	0.196	0.218	0.647
	*Error (Session)*	17	0.901		
	CONDITION*SESSION	1	0.050	0.246	0.626
	Error (CONDITION * SESSION)	17	0.204		
dACC (ROI-2) [Glx/tCr]	**CONDITION**	**1**	**0.040**	**6.075**	**0.025**
	*Error (CONDITION)*	17	0.007		
	SESSION	1	0.018	0.672	0.424
	*Error (Session)*	17	0.027		
	CONDITION*SESSION	1	0.000	0.028	0.870
	Error (CONDITION * SESSION)	17	0.004		
dACC (ROI-2) [tCr]	CONDITION	1	0.395	0.939	0.346
	*Error (CONDITION)*	17	0.420		
	SESSION	1	0.009	0.004	0.947
	*Error (Session)*	17	1.932		
	CONDITION*SESSION	1	0.300	1.331	0.265
	Error (CONDITION * SESSION)	17	0.226		

*(df = degrees of freedom; MS = Mean Squares). The bold values indicate a significant effect (p < 0.05)*.

**Table 3:** Mean Glx/tCr for REST and PAIN conditions and ΔGlx/tCr across combined and individual sessions in the dACC (ROI-2).

	**REST [Glx/tCr]**	**(SD)**	**(SE)**	**PAIN [Glx/tCr]**	**(SD)**	**(SE)**	**Mean difference (95 CI)**	**Mean change (%)**
Sessions combined	1.04	0.13	0.02	1.08	0.17	0.03	0.05 (CI 0.01, 0.09)	+4.52
Session A	1.06	0.14	0.03	1.10	0.18	0.04	0.05 (−0.00, 0.09)	+4.26
Session B	1.02	0.12	0.03	1.07	0.17	0.04	0.05 (−0.02, 0.11)	+4.88

*[Glx/tCr], Glx (glutamate + glutamine) scaled to total creatine; SD, Standard Deviation; SE, Standard Error*.

For ΔGlx/tCr in the dACC ROI between the averaged blocks of REST and PAIN there was poor test-retest reliability (ICC = 0.272, %CV = 10.5%) and limited reproducibility (VAR = 10.3). There was no significant relationship between ΔGlx/tCr in the first and second sessions (*r* = 0.264, *p* = 0.289).

For Glx/tCr in the ACC (ROI-1), there was no significant CONDITION ^*^ SESSION interaction [*F*_(1,17)_ = 0.202, *p* = 0.659] and no significant main effects of CONDITION [*F*_(1,17)_ = 2.620, *p* = 0.124] or SESSION [*F*_(1,17)_ = 2.703, *p* = 0.119] (Table 2).

Exploratory analyses with a three-way repeated measures ANOVA, examining Glx/tCr across individual blocks, demonstrated a significant main effect of CONDITION for Glx/tCr in the dACC [*F*_(1,17)_ = 10.407, *p* = 0.005] with no significant interactions (i.e., BLOCK^*^CONDITION, BLOCK ^*^ SESSION, CONDITION^*^ SESSION or BLOCK^*^CONDITION^*^SESSION) and no significant main effects of BLOCK or SESSION (Supplementary Table 1). For Glx/tCr in the ACC (ROI-1) there were no significant interactions and no significant main effects of BLOCK, CONDITION or SESSION for the repeated measures ANOVA (Supplementary Table 1).

Uncorrected tCr signal values were found to be stable for averaged REST and PAIN conditions and across sessions with no significant CONDITION ^*^ SESSION interactions and no significant main effects of CONDITION or SESSION for either ROI (Table 2). Furthermore, tCr signal values remained stable across individual blocks of REST and PAIN (Supplementary Figure 2). Three-way repeated measures ANOVAs revealed no significant interactions and no main effects of BLOCK, CONDITION or SESSION on tCr values in either ROI (Supplementary Table 1).

### Tissue Segmentation and SNR

For the ACC ROI, the mean ± standard deviation tissue proportions were 65 ± 4% GM, 10 ± 3% WM, 25 ± 6% CSF and 65 ± 4% GM, 10 ± 3% WM, 25 ± 5% CSF for session A and B respectively. For the dACC ROI, the mean tissue proportions were 63 ± 4% GM, 21 ± 6% WM, 16 ± 5% CSF and 62 ± 4% GM, 21 ± 6% WM, 16 ± 5% CSF for sessions A and B, respectively. The relatively small spread of proportions indicates consistency of voxel position across subjects. Furthermore, there were no significant differences in GM, WM and CSF tissue proportions between sessions indicating consistency of voxel positioning across scans (Supplementary Table 2). Example voxel positioning and corresponding ^1^H-fMRS spectra are shown in Figures 3A,B.

For total averaged block conditions, mean SNR for the ACC ROI was 31.4 ± 5.7 for REST and 31.5 ± 5.8 for PAIN. For the dACC ROI, these values were 34.9 ± 5.0 and 35.4 ± 4.6 for REST and PAIN conditions, respectively. There were no significant differences in SNR between REST and PAIN conditions in either ROI (Supplementary Table 3).

### Relationship Between Glx/tCr Changes and Pain VAS Scores

Correlations between ΔGlx/tCr values and pain VAS are shown in Table 4. There were no consistent significant associations between ΔGlx/tCr (PAIN–REST) conditions and pain VAS for all blocks combined across each scanning session.

**Table 4:** Correlations with changes in Glx/tCr across REST and PAIN conditions and pain VAS.

	**VAS**	***p***
**Session A**		
ROI-1 (ACC) Δ [Glx/tCr]	0.38	0.12
ROI-2 (dACC) Δ [Glx/tCr]	0.14	0.58
**Session B**		
ROI-1 (ACC) Δ [Glx/tCr]	−0.09	0.72
ROI-2 (dACC) Δ [Glx/tCr]	0.15	0.56

## Discussion

In this study we utilised ^1^H-fMRS at 3T in a test-retest design to examine dynamic changes in Glx/tCr in response to acute pressure pain stimulation within the ACC and dACC. The pain paradigm elicited a statistically significant increase in dACC Glx/tCr overall across both scanning sessions. However, these changes were associated with limited within-subject reliability. Overall, these results indicate that this paradigm does elicit increases in dACC Glx/tCr, but the reliability of this effect may be insufficient for some future applications.

The cingulate cortex is functionally heterogenous (28) and has been shown to be involved in a range of functions related to pain including the perception of pain, attention to pain and emotional processing of pain (27, 36, 37). Several previous studies have utilised ^1^H-fMRS to investigate changes in glutamate measures in ACC/dACC regions during painful stimuli in healthy volunteers (20–22, 24, 38, 39), however none have reported the associated test-retest reliability. While some studies have reported significant increases in Glu (20, 22), Glx (20, 22) and Glx/tCr (21), others have found no significant changes in glutamate (38, 39) or reductions in glutamate during noxious stimulation (24). The significant increase in dACC Glx/tCr we observed in our pressure pain paradigm across combined scanning sessions (4.5%) is lower in magnitude than the increase in Glx/tCr (21.5%) previously reported during heat stimuli in an event related design (21). The lack of any significant findings in the ACC ROI in our study could be explained by our choice of pain stimulus that did not reliably engage this area. When a similar pressure-pain paradigm was used in a fMRI study, peak BOLD coordinates during noxious stimulation were not observed in the ACC, but instead more posterior cingulate cortex regions (26). Although our dACC voxel was similarly positioned to that used in the studies by Cleve et al. (21) and Archibald et al. (22), the less robust Glx/tCr change in our study may be explained by the pain stimulus used (pressure rather than heat), or differences in acquisition parameters, including T1 and T2 weighting, that might have also contributed. While Archibald et al. (22) acquired interleaved non-water suppressed spectra, allowing absolute metabolite quantification, in our study the water unsuppressed acquisitions were non-interleaved, acquired at the end of the sequence, necessitating the use of tCr scaling that may have led to a relative underestimate of Glx signal change. It should be noted that in the study by Cleve et al. (21), only female participants were included and Archibald et al. (22) reported nominally larger increases in glutamate and Glx in response to pain in females. Our study was not powered to detect metabolite ratio differences between genders, however future ^1^H-fMRS research should include gender-based analyses to better understand potential differences in glutamatergic signalling between the sexes.

**Figure 5:**
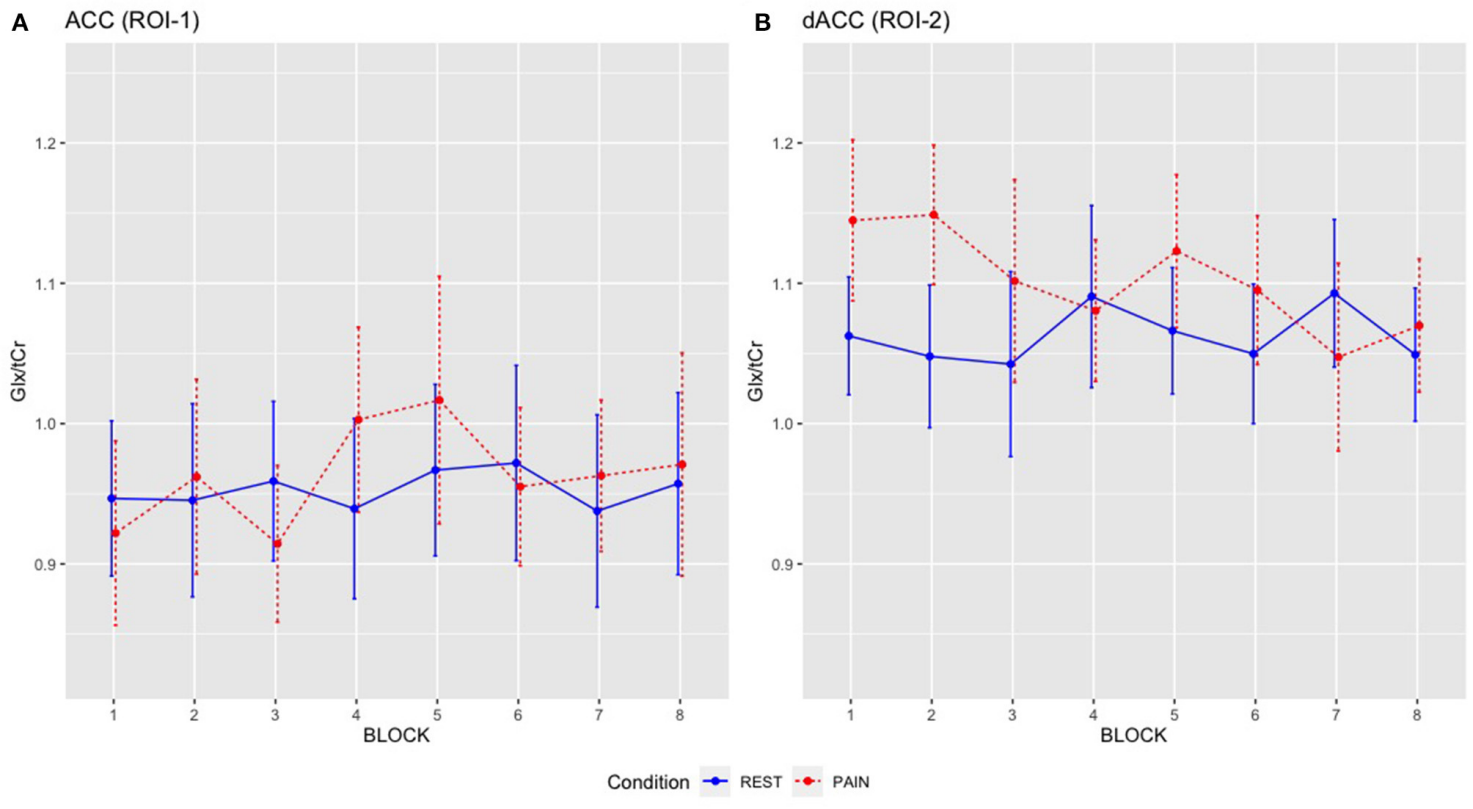
Mean Glx/tCr for individual REST and PAIN blocks across scanning sessions: **(A)** ACC Glx/tCr, **(B)** dACC Glx/tCr. REST condition is shown in blue solid line and PAIN condition is shown in red dashed line. Error bars represent the 95% CI.

Although there was no significant BLOCK by CONDITION interaction for dACC Glx/tCr, qualitative inspection of the data indicated diminishment of Glx/tCr responses on repeated stimulation (Figure 5), which may be of neurobiological interest as well as presenting a practical challenge for future ^1^H-fMRS studies. This pattern of reducing dACC Glx/tCr across pain blocks suggests potential adaptation/ habituation effects may be occurring within the glutamate system on repeated stimulation (7). This is comparable with findings from Archibald et al. (22) that demonstrated increased concentrations in glutamate measures at the onset of pain but continued painful stimulus was not accompanied by corresponding changes in glutamate or Glx. While increasing the quantity of painful stimuli has the potential to increase power, if the stimuli are repetitive (and predictable) then a repetition suppression effect may be present, accompanied by reductions in glutamate (40). In agreement with Archibald et al. (22), we found no relationship between pain ratings and Glx/tCr concentrations. Together, this supports a hypothesis that greater increases in dACC glutamate measures may be involved in the initial detection of novel noxious stimuli but less so in the tracking of repeated painful stimuli. Indeed, it has been proposed that an extensive cortical network that is particularly responsive to nociceptive stimuli (including the cingulate, somatosensory, insular, frontal and parietal areas), often referred to as the “pain matrix,” may reflect a salience detection system (41). This network could reflect a mechanism through which significant events for the body's integrity are detected, acting as a defensive system, alerting to potential damaging or dangerous threats (41). Therefore, the context within which the painful stimuli appear is key. With an experimentally evoked painful stimulus such as ours, participants implicitly know they will not come to any harm, which contrasts with prolonged and inescapable pain seen in chronic pain patients or other models.

The precise mechanisms underlying increases in Glx/tCr in response to painful stimulation and detected by ^1^H-fMRS are not yet clear. One potential mechanism could involve increased metabolic turnover during activation through various pathways including increased glutamate/glutamine cycling, increased energy production rate through the tricarboxylic acid cycle (TCA) (12, 42) and increased flux through the malate-aspartate shuttle (MAS) (9, 43). Another speculative theory of “compartmental shifts” proposes that glutamate may be less or more MRS visible, depending on its location within vesicles or within synaptic, extracellular and astrocytic pools, respectively and on MRS acquisition parameters (7, 8, 44). Further studies using ^13^C MRS (45) and at higher field strengths to separate glutamate and glutamine are required to understand these mechanisms further.

Strengths of our study include the application advantages of our noxious pressure paradigm including practicality, ability to control pain level (tailored to individual thresholds) and reliability of self-reported pain measures as previously demonstrated (26). Furthermore, to our knowledge, this is the first ^1^H-fMRS pain study to report on test-retest reliability across separate imaging sessions. One limitation to this study is the low sample size (*n* = 18). Although this is comparable to previous studies [*n* = 18 (22), *n* = 20 (21), *n* = 12 (20)], power was limited by the relatively smaller effect sizes for ΔGlx/tCr that were observed in our paradigm, with the effect of condition not reaching significance during the first session alone. Larger sample sizes are recommended for future studies which aim to investigate between-subjects differences in a single scanning session using this paradigm. Despite advantages associated with the pressure pain paradigm employed here, there are also limitations which may explain the less robust glutamate measure change. Although block-design analyses were employed with a view to increase statistical power, the pressure stimuli were not continuous throughout the PAIN block. Although this design was chosen to make the experiment more tolerable for participants, glutamate signal may have decreased when the stimuli were not present. To achieve more robust, reliable increases in glutamate measures, applying the pressure stimuli for a longer duration with less repetitions may be preferable. As mean pain VAS scores were higher for the second run of the paradigm (dACC) than the first run (ACC), this suggests a potential effect of sensitisation. Since the experimental order for the ROIs was fixed, we cannot exclude potential order effects and the possibility that pain-induced dACC Glx/tCr changes may have been lesser (or ACC Glx/tCr greater) if the acquisition order was reversed. The T_E_ of 105 ms was chosen based on a previous glutamate ^1^H-fMRS study (40), however, this T_E_ is quite long with significant J-modulation and attenuation due to T2 relaxation and therefore may not have been most optimal. Future ^1^H-fMRS work should directly compare long and short T_E_ times to assess reliability of glutamate/glutamine/Glx measures. Finally, acquired ^1^H-fMRS signals were not corrected for tissue type or relaxation differences and instead were referenced to tCr which reduces the need for tissue content correction. Although there were no significant differences in tissue proportions between scan sessions it is possible detected metabolite ratio changes may have been influenced by small differences in voxel tissue content.

## Conclusions

As glutamatergic signalling is increasingly implicated across a range of psychiatric, neurological and pain disorders there is a need to develop reliable methodologies for probing the glutamate system and understanding glutamate dynamics. Such tools may ultimately be used to develop further understanding of biological illness mechanisms and support in the development of glutamate-acting drugs. While our pressure-pain ^1^H-fMRS paradigm demonstrated the feasibility of detecting increases in Glx/tCr associated with pressure-pain in the dACC across combined scanning sessions, the magnitude was variable within-subjects and was not sustained across the entire paradigm. We recommend alternative stimuli, paradigms and acquisition parameters are tested in future test-retest ^1^H-fMRS studies to determine more reliable methodologies for probing glutamate dynamic responses in patient groups and future experimental medicine studies.

## Data Availability Statement

The raw data supporting the conclusions of this article will be made available by the authors, without undue reservation.

## Ethics Statement

The studies involving human participants were reviewed and approved by King's College London college ethics committee (Reference HR-18/19-8825). The patients/participants provided their written informed consent to participate in this study.

## Author Contributions

LJ, DL, JJ, MH, JS, and AE contributed to the conception and design of the study. LJ collected the data and performed the data analysis, supervised by AE and JS. LJ wrote the first draft of the manuscript. All authors provided critical feedback, contributed to manuscript revision, and approved the submitted version.

## Author Disclaimer

The views expressed are those of the authors and not necessarily those of the NHS, the NIHR, or the Department of Health.

## Conflict of Interest

JS: In the last 3 years, he has been PI or sub-investigator on studies sponsored by Takeda, Janssen and Lundbeck Plc. He has attended an Investigators' meeting run by Allergan Plc. The remaining authors declare that the research was conducted in the absence of any commercial or financial relationships that could be construed as a potential conflict of interest.

## Publisher's Note

All claims expressed in this article are solely those of the authors and do not necessarily represent those of their affiliated organizations, or those of the publisher, the editors and the reviewers. Any product that may be evaluated in this article, or claim that may be made by its manufacturer, is not guaranteed or endorsed by the publisher.
