# Supplementary material for: Imaging Brain Glx Dynamics in Response to Pressure Pain Stimulation: A 1H-fMRS Study
Source: Front Psychiatry. 2021 Jul 28;12:681419. doi: 10.3389/fpsyt.2021.681419 (PMC8357306; doi:10.3389/fpsyt.2021.681419)
Supplement: Supplementary file 1 [file Data_Sheet_1.PDF]

SUPPLEMENTARY MATERIAL

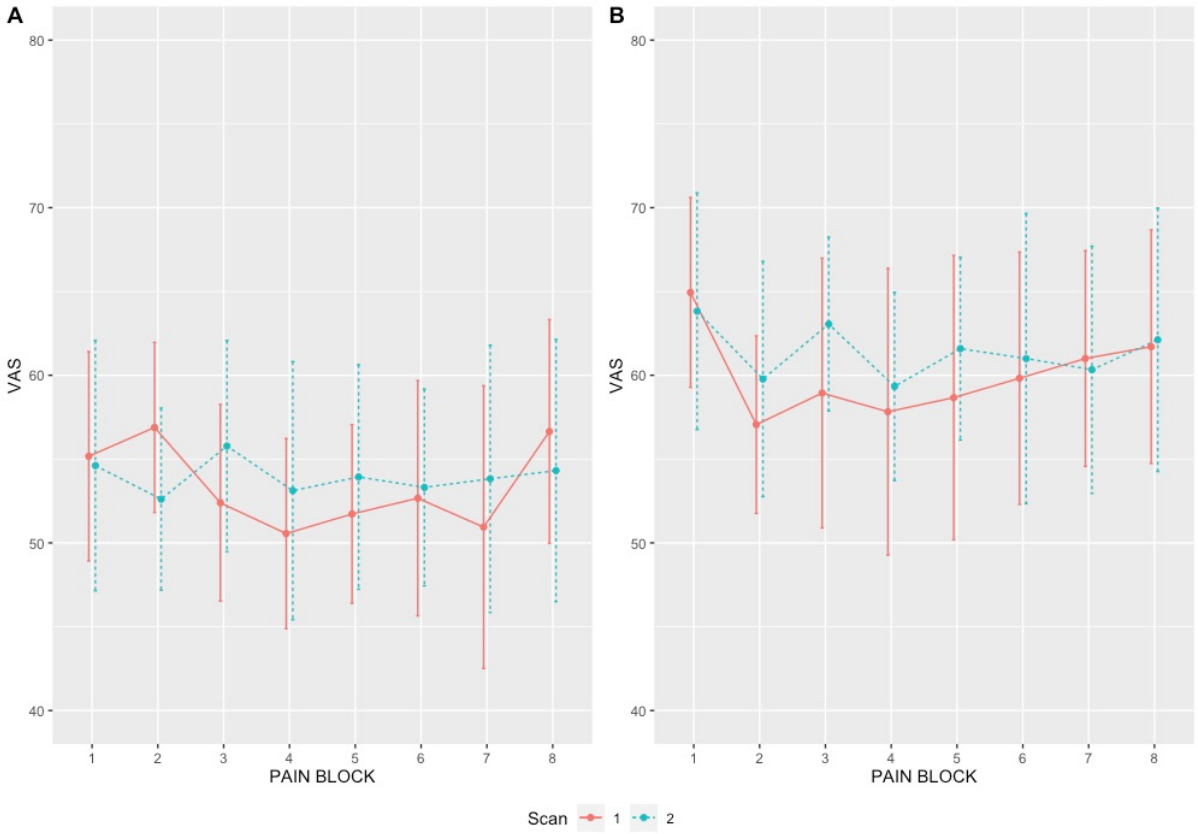

Figure S1: Mean pain VAS scores for individual pain blocks: (A) ACC (ROI-1) (B) dACC (ROI-2). Session A is shown in red solid line and session B shown in blue dashed line. Error bars represent the 95% CI. Mean pain VAS scores (across all blocks) were numerically higher for the second run (dACC (ROI-2)) than the first (ACC (ROI 1)) for both sessions A and B. For session A this did not reach statistical significance (mean difference = 6.60 (-0.97, 14.17), t(17) = 1.839, p = 0.083). However, for session B this difference was statistically significant (mean difference = 7.49 (1.91, 13.08), t(17) = 2.83, p = 0.012).

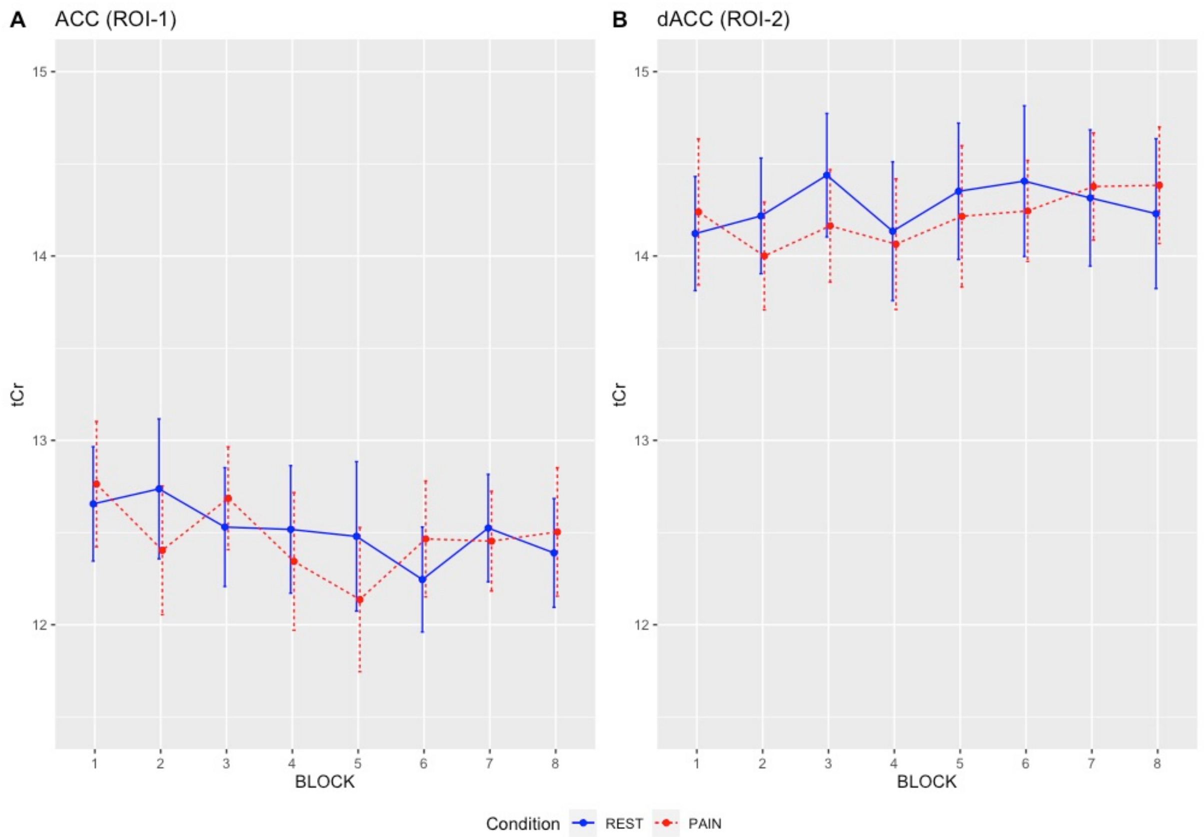

Figure S2: Mean tCr signal values across individual blocks of REST and PAIN across scanning sessions: (A) ACC (ROI-1) (B) dACC (ROI-2). REST condition is shown in blue solid line and PAIN condition is shown in red dashed line. Error bars represent the 95% CI.

Table S1: Three-way ANOVA Summary Table for Glx/tCr and tCr for each ROI

ROI & Metabolite	Source	df	MS	F	p
**ACC (ROI-1) [Glx/tCr]**	BLOCK	7	0.025	0.631	0.729
	Error (BLOCK)	119	0.040		
	CONDITION	1	0.015	0.302	0.590
	Error (CONDITION)	17	0.051		
	SESSION	1	0.252	3.287	0.088
	Error (Session)	17	0.077		
	*BLOCK*CONDITION*	7	0.025	0.800	0.589
	*Error (BLOCK*CONDITION)*	119	0.031		
	*BLOCK*SESSION*	7	0.032	0.984	0.446
	*Error (BLOCK*SESSION)*	119	0.032		
	CONDITION*SESSION	1	0.098	4.096	0.059
	*Error (CONDITION*SESSION)*	17	0.024		
	*BLOCK*CONDITION*SESSION*	7	0.020	0.560	0.787
	*Error (BLOCK*CONDITION*SESSION)*	119	0.036		
**ACC (ROI-1) [tCr]**	BLOCK	7	1.287	1.466	0.186
	Error (BLOCK)	119	0.878		
	CONDITION	1	0.233	0.321	0.579
	Error (CONDITION)	17	0.727		
	SESSION	1	3.818	0.770	0.392
	Error (Session)	17	4.956		
	*BLOCK*CONDITION*	7	0.895	1.290	0.261
	*Error (BLOCK*CONDITION)*	119	0.694		
	*BLOCK*SESSION*	7	0.848	0.890	0.516
	*Error (BLOCK*SESSION)*	119	0.953		
	CONDITION*SESSION	1	0.617	0.756	0.397
	*Error (CONDITION*SESSION)*	17	0.816		
	*BLOCK*CONDITION*SESSION*	7	0.232	0.314	0.946
	*Error (BLOCK*CONDITION*SESSION)*	119	0.738		
**dACC (ROI-2) [Glx/tCr]**	BLOCK	7	0.018	1.178	0.320
	Error (BLOCK)	119	0.015		
	**CONDITION**	**1**	**0.216**	**10.407**	**0.005**
	Error (CONDITION)	17	0.021		
	SESSION	1	0.176	1.163	0.296
	Error (Session)	17	0.151		
	*BLOCK*CONDITION*	7	0.042	1.729	0.109
	*Error (BLOCK*CONDITION)*	119	0.024		
	*BLOCK*SESSION*	7	0.011	0.519	0.818
	*Error (BLOCK*SESSION)*	119	0.022		
	CONDITION*SESSION	1	0.023	1.347	0.262
	*Error (CONDITION*SESSION)*	17	0.017		
	*BLOCK*CONDITION*SESSION*	7	0.018	0.716	0.659
	*Error (BLOCK*CONDITION*SESSION)*	119	0.025		
**dACC (ROI-2) [tCr]**	BLOCK	7	0.709	1.127	0.351
	Error (BLOCK)	119	0.629		
	CONDITION	1	0.623	0.346	0.564
	Error (CONDITION)	17	1.799		
	SESSION	1	2.800	0.267	0.612
	Error (Session)	17	10.484		
	*BLOCK*CONDITION*	7	0.460	0.708	0.665
	*Error (BLOCK*CONDITION)*	119	0.650		
	*BLOCK*SESSION*	7	0.192	0.242	0.974
	*Error (BLOCK*SESSION)*	119	0.796		
	CONDITION*SESSION	1	0.211	0.240	0.631
	*Error (CONDITION*SESSION)*	17	0.878		
	*BLOCK*CONDITION*SESSION*	7	0.458	0.754	0.627
	*Error (BLOCK*CONDITION*SESSION)*	119	0.607		

(df = degrees of freedom; MS = Mean Squares)

Table S2: Mean voxel tissue composition for each ROI for each scan session

	Session A, Mean (SD)	Session B, Mean (SD)	P
**ACC (ROI-1)**			
**GM**	0.65 (0.04)	0.65 (0.04)	1.00
**WM**	0.10 (0.03)	0.10 (0.03)	0.29
**CSF**	0.25 (0.06)	0.25 (0.05)	0.49
**dACC (ROI-2)**			
**GM**	0.63 (0.04)	0.62 (0.04)	0.22
**WM**	0.21 (0.06)	0.21 (0.06)	0.62
**CSF**	0.16 (0.05)	0.16 (0.05)	0.42

Abbreviations: GM: Grey matter; WM: white matter; CSF: cerebrospinal fluid; **P**: P-value pairwise comparisons (alpha=0.05, two-tailed).

Table S3: Mean SNR values

	REST, mean SNR (SD)	PAIN, mean SNR (SD)	P
**ACC (ROI-1) (All blocks)**	31.38 (5.67)	31.51 (5.80)	0.67
**dACC (ROI-2) (All blocks)**	34.92 (4.95)	35.38 (4.61)	0.12

P: P-value pairwise comparisons (alpha=0.05, two-tailed).
